# A joint survival model for estimating the association between viral load outcome and survival time to death among HIV/AIDS patients attending health care and treatment centers in Tanzania

**DOI:** 10.1186/s12889-023-16977-x

**Published:** 2023-10-25

**Authors:** Habiel Benjamin Luvanda, Elevatus Nkebukwa Mukyanuzi, Rocky R. J. Akarro

**Affiliations:** 1 Department of Mathematics and Statistics, University of Dodoma, Dodoma, Tanzania; 2 Department of Statistics, University of Dar es Salaam, Dar es Salaam, Tanzania

**Keywords:** HIV/AIDS, Mortality, Cox proportional hazards model, Linear mixed model, Joint survival model

## Abstract

**Background:**

Globally, HIV/AIDS is one of the diseases that have a huge burden in terms of cost and health of individuals; and Sub-Sahara Africa is the highly affected region by the pandemic. Tanzania is among the countries that have a higher prevalence of HIV/AIDS-related mortality. This study aimed at using the joint survival model to estimate the association between viral load outcome and survival outcome to death adjusting for age, sex, adherence, and visit date.

**Methods:**

Secondary data from a retrospective cohort of HIV patients attending health care and treatment centers were used to analyze the association between the longitudinal viral load and time-to-death outcomes. The three-step analysis was based on the individual mixed effects linear model and the Cox proportional hazards models to estimate the significance of the independent outcomes, and the joint survival model as a final model. The joint model was used to estimate the factors affecting the average change in log viral load over time and the risk factors for the survival time of HIV patients. The exposures for both models were ART adherence status, age, male, and visit date whereas the outcome for the LMM was log viral load and the outcome for the Cox PH model was time-to-death in years.

**Results:**

The joint survival model results revealed that a 10-year increase in age was associated with a 37% increased risk of death (HR = 1.369, 95% CI: 1.253–1.844), and being male was associated with a 49% higher risk of death (HR = 1.489, 95% CI: 1.202–1.844) compared to females. The results also provided evidence of an association between the longitudinal log viral load and the survival time to death ) whereby a unit increase in the log viral load was associated with a 26% increase in the risk of death (HR = 1.262, 95% CI: 1.226–1.301).

**Conclusion:**

The joint survival model analysis provided valuable insights into the associations between time to death and log viral load with adherence to ART, age, visit date, and sex of the patients. This implies that viral load suppression, as well as sex and age-specific interventions, are necessary for reducing HIV/AIDS-related deaths.

## Background

Globally, HIV/AIDS is one of the diseases which have a huge burden in terms of cost. For example, more than US$500 billion was estimated to be spent on the prevention, care, and treatment of HIV/AIDS globally between 2000 and 2015 [1, 2]. Moreover, the HIV/AIDS disease is a burden in terms of health aspects of individuals; for instance, by 2018 there were over 39 million HIV/AIDS-related deaths and more than 36 million people living with HIV [2–4]. Despite the much lower cases in Northern Africa and the Middle East, Sub-Saharan Africa is still the most highly affected region by the HIV/AIDS pandemic. For instance, by the year 2017 it was estimated that in SSA, there were on average 1.2 million new HIV infection cases and about 712,000 HIV-related deaths. This means a lot in terms of new patients who might need antiretroviral (ARV) therapy in the region [2].

In Tanzania, the Tanzania HIV Impact Survey (THIS) 2016–2017 revealed that the prevalence of HIV among individuals aged 15 to 49 years was 4.7% with an annual incidence of 0.27% [5]. Nevertheless, the youth category aged 25 to 34 years is the one at more risk population category with an annual incidence of 0.44%, which is above the national annual incidence [5]. Moreover, it is observed that the key populations such as female sex workers, prisoners, people who inject drugs, homosexual men, and transgender persons are at higher risk of contracting the disease than any other persons in the population [6].

Several studies have explored the survival outcome of HIV/AIDS patients using Kaplan-Meir and proportional hazards models [7–12]. In studying the association between the repeated measures of the biomarker and the survival outcomes, joint survival models have proven to be more efficient and several studies in Sub-Saharan Africa have used the longitudinal CD4 as a longitudinal trajectory and survival outcome [13–16]. However, there are limited studies in Tanzania that have examined the joint outcomes of viral load trajectory and survival outcome as most of the studies have identified only one outcome. Furthermore, studies that have been conducted in other countries that used a joint model have used CD4 count as the longitudinal trajectory. This study aims to examine the association between the changes in the longitudinal trajectory of the biomarker, in this case, the log of HIV viral load and the risk of death of the patients by jointly modeling the two outcomes while adjusting for ART adherence status, age, visit date, and sex. In this study, the viral load outcome has been used instead of CD4 counts to examine the effect of viral load suppression on survival outcome.

## Methods

### Data source

Secondary data from the National AIDS Control Programme (NACP) for HIV-positive patients attending health care and treatment clinic was used.

### Study design, study area, and participant inclusion criteria

The study employed a retrospective longitudinal study design. The patient visits data from 1996 to 2020 were used to generate the longitudinal data with the earliest observation being in September 1996 and the latest being observed in November 2020. The analyzed data were from Tanzania’s mainland. Patients aged 15 years and over were selected to be included in the study, whereas patients under the age of 15 were excluded because the study was confined to adults.

### Variables of the study

The outcome variables were logarithmic transformed viral load as a longitudinal trajectory and time to death event in years as a survival outcome. The covariates used to predict the outcomes were age group in years, male sex, patient visit date, and adherence status. These variables were included to determine their effects on the outcome variable. The variable ART regimen was not included because it was not available in the dataset, but its effect can be observed through the viral load. The description of the study variables is elaborated in Table 1 below. The variable viral load was logarithmically transformed for manageability and normalization (minimum viral load was 11 copies/mL and the maximum value was $2.57\times {10}^{18}$ copies/mL).

**Table 1:** Variables of the study

Variable type	Variable name	Variable values	Definition
Outcome Variables	Log viral load	2.398, ………, 42.39	Continuous
	Death status	0 = Censored, 1 = Died	Categorical
	Survival time (years)	0, 1, 2, ……, 24	Continuous
Covariates	Adherence status	0 = Good, 1 = Poor	Categorical
	Male sex	0 = No, 1 = Yes	Categorical
	Age group (years)	1 = 15–24, ….	Categorical
	Visit date	Date	Continuous

### Data processing, analysis, and presentation methods

Data processing, management, presentation, and analysis were performed by using STATA. Separate analyses for proportional hazards survival and linear mixed effects were performed to estimate the survival and longitudinal biomarker outcomes respectively. Finally, a joint survival model that combines both the time-to-death survival and the log viral load as longitudinal outcomes was performed. The joint survival model is constructed in three steps which are the proportional hazards (PH) model for the time to event, the linear mixed model (LMM) for the longitudinal trajectory, and the joint survival model that combines both the PH and LMM models.

#### The cox proportional hazards model

A Cox PH model with a time-varying covariate of the observed viral load biomarker ${y}_{i}\left(t\right)$ for the ${i}^{th}$ patient at time $t$ is as follows:

1 $$\begin{gathered}{h_i}\left( t \right) = {h_o}\left( t \right)\exp {[^{}}_{}\gamma + {\lambda _1}sex + {\lambda _2}age + {\lambda _3}adherence + \\ {\lambda _4}visitdate + \gamma {y_i}\left( t \right)] \\ \end{gathered}$$

But this model assumes that the viral load biomarker doesn’t change until a new measurement is taken, as such, it ignores measurement error in the biomarker.

#### The linear mixed model

The longitudinal trajectory outcome model assumes the observed continuous biomarker of the log viral load denoted as ${y}_{i}\left(t\right)$ and is defined as follows:

2 $${y}_{i}\left(t\right)={m}_{i}\left(t\right)+{e}_{i}\left(t\right),$$

Where:

3 $$\begin{gathered}{m_i}\left( t \right) = {\beta _0} + {\beta _1}sex + {\beta _2}age + {\beta _3}adherence + \\ {\beta _4}visitdate + ({X_i}|patient) \\ \end{gathered}$$

In this model, $${m}_{i}\left(t\right)$$ is called the trajectory function for the true unobserved value of the viral load biomarker for the $${i}^{th}$$ patient at time $${t}$$, $${X}_{i}\left(t\right)$$$${X}_{i}$$ is the $${n_i} \times q$$ observed design matrix corresponding to the covariates of the random effects (repeated measures of the patients), $${\beta }_{i}$$ are parameter estimates, and $${e}_{i}$$ is the $${n}_{j}\times 1$$ vector of residuals for response variable.

The assumption for this model is ${e}_{i}\sim N(0,{\Sigma } )$; where ${\Sigma }$ is the variance-covariance matrix for ${e}_{i}$ for outcome variable.

#### The joint survival model

The true unobserved longitudinal profile up to time $t$ is defined as ${\text{M}}_{\text{i}}\left(\text{t}\right)=\left\{{\text{m}}_{\text{i}}\right(\text{s}); 0 \le \text{s} \le \text{t}\}$. Therefore, a joint survival model is defined by linking the component models for the linear mixed model and survival sub-model as follows [17]:

4 $$\begin{gathered} h\left\{ {t|{M_i}\left( t \right),{X_i}} \right\} = {h_o}\left( t \right){\text{exp}}[{\lambda _0} + {\lambda _1}sex + {\lambda _2}age + {\lambda _3}adherence + \\ {\lambda _4}visitdate + \gamma {m_i}\left( t \right)] \\ \end{gathered}$$

where ${h}_{0}\left(t\right)$ is the baseline hazard function, and ${X}_{i}$ a set of baseline time-independent covariates with an associated vector of log hazard ratios, ${\lambda }_{i}$.

## Results

### Demographic and clinical characteristics of the patients

As observed in Table 2, this study involved a total of 1,134,723 patients, with 32.4% being males and 67.6% being females. The majority of the patients were good adherent to ART at 97.8% and only 2.2% were poor adherent to ART. The majority of the patients were in the 25–34, 35–44, and 45–54 age groups at 31.14%, 33.34%, and 17.46% respectively while few patients were in the 15–24, 55–64, and 65 + age groups at 9.33%, 6.51%, and 2.22% respectively. The mean value of the log of viral load was 3.55, with a minimum value of 2.4 and a maximum value of 42.39. These log viral load values varied throughout time. In the survival analysis, there were 20,983 death outcomes with a minimum survival time to death of 0 years, a maximum survival time to death of 24 years, and a median survival time of 3 years.

**Table 2:** Demographic and clinical characteristics of the patients

Variable	Frequency	Percent		Missing	
Male sex					
Yes	368,090	32.400			
No	766,633	67.600			
**Adherence Status**					
Good (≥95% of adherence)	819,009	97.760	296,907		
Poor (<95% of adherence)	18,807	2.240			
**Age group**					
15–24	105,919	9.330			
25–34	353,343	31.140			
35–44	378,282	33.340			
45–54	198,121	17.460			
55–64	73,909	6.510			
65 and above	25,149	2.220			
**Death status**					
Number of died subjects	20,983				
**Log viral load**					
Number of observations	113,175				
Minimum	2.400				
Maximum	42.390				
Mean	3.550				
**Survival Time to Death (Years)**					
Minimum	0				
Maximum	24				
Median	3				
**Total Observations (N)**	**1,134,723**	**100**			

### The results of a linear mixed effects model for the log viral load outcome

The outcome of interest was the continuous logarithm-transformed viral load count. The linear mixed model accounts for fixed and random effects, and missing values and allows for unequal spaced time intervals. The mixed effects model was run for 103,536 observations of patients which were nested in 99,433 groups of individual patients with repeated measures.

The mixed effects model results in Table 3 show that being male was associated with an increase in the log viral load by 0.27, and for 10 years increase in age the log of viral load decreased by 0.127. Likewise, an increase in patient visit dates was associated with a decrease in the log viral load by 0.0008.

**Table 3:** The adjusted linear mixed effects model for the factors associated with patients’ log viral load

	Coefficient (95% CI)	Std. error	P-value
Adherence status	1.414 (1,324, 1.503)	0.046	< 0.001
Male sex	0.270 (0.239, 0.302)	0.016	< 0.001
Age group	-0.127 (-0.139, -0.115)	0.006	< 0.001
Visit date	-0.0008 (-0.0008, -0.00077)	0.000	0.001
Intercept	21.244 (20.29, 21.99)	0.383	< 0.001

Note: Std. error = standard error

The intra-class correlation (ICC) post-estimation test result for LMM was 0.388. This shows that the hierarchical LMM was an appropriate model and that 39% of the variability in the log viral load was explained by variables and clustering by repeated measures of the patient.

### The Cox proportional hazard model results

The Cox PH analysis is a commonly used statistical technique to assess the association between risk factors and survival outcomes. The relationship between HIV viral load and patients’ mortality has been extensively studied, as it plays a crucial role in determining the progression and management of HIV/AIDS. In this model, the findings of a Cox proportional hazards (PH) analysis focused on examining the independent effect of adherence status, male sex, age group, and calendar time (visit date) on the risk of death. The variable visit date was excluded from the analysis because it resulted in the violation of the proportional hazards assumption. The Cox PH model was employed to determine the independent effect of HIV viral load on the risk of death while controlling for other relevant factors. The proportional hazard model results showed that, at a 1% level of significance, adherence status, male sex, age group, and visit date were significantly associated with patients’ survival outcome.

The Cox PH results as shown in Table 4 revealed that poor adherence to ART was associated with a 21% higher risk of patients’ death independent of sex, and age group as compared to good adherence to ART. Being male was associated with a 65% higher risk of death as compared to female patients. Likewise, a higher age group was associated with a higher risk of death as 10 years increase in age was associated with a 17% risk of dying.

**Table 4:** A Cox proportional hazards model results for factors associated with patients’ death over time

	Hazard ratio (95% CI)	Std. error	P-value
Adherence status	1.21 (0.115, 1.326)	0.054	< 0.001
Male sex	1.646 (1.601, 1.692)	0.023	< 0.001
Age group	1.166 (1.153, 1.180)	0.007	< 0.001

Outcome variable: survival time to death (years)

The post-estimation test for the proportional hazards assumption indicated the proportional hazards assumption was not violated at a 1% level of significance since the Chi-square test statistic was not significant (P = 0.089). Therefore, the Cox proportional hazards model was appropriate for the analysis of time-to-event data.

### The joint survival model results

The joint survival model is a statistical approach that allows for the simultaneous analysis of time-to-event outcomes and longitudinal data. In this study, researchers utilized a joint survival model to estimate the relationships between time to death and log viral load adjusting for adherence status, male sex, age group, and patient visit date. The parameter estimates and corresponding confidence intervals were derived to quantify these associations. The hazard rates for the survival part were obtained by exponentiating the parameter estimates.

The results of the joint survival model estimates as observed in Table 5 show that a unit increase in the log of viral loads resulted in an increase in the risk of death for the patient by 26% (HR = 1.262, 95% CI: 1.226, 1.307). The survival part hazards estimates for the age group showed that a 10-year increase in age resulted in a 37% increase in the risk of death (HR = 1.369, 95% CI: 1.253–1.495). Being male was associated with a 49% higher risk of dying as compared to female patients (HR = 1.489, 95% CI: 1.202–1.844). The results also show that adherence status and visit date were not significant in the survival part of the joint model.

For the longitudinal part, all variables were significant at a 1% level of significance. Being poor adherent to ART was associated with 1.234 increase in the log viral load (b = 1.234, 95% CI: 1.226, 1.242), being male was associated with an increase in the log viral load by 0.228 (b = 0.228, 95% CI: 0.225, 0.230), and for 10 years increase in age the log viral load was decreased by 0.118 (b=-0.118, 95% CI: -0.119, -0.117). A day change in visits was associated with a decrease in the log viral load by 0.001 (b=-0.00072, 95% CI: -0.00073, -0.00071).

**Table 5:** The joint survival model estimates for factors associated with patients’ log viral load and time to death

	Parameters	Std. error	P-value
**Longitudinal**	**Coefficient (95% CI)**		
Adherence status	1.234 (1.226, 1.242)	0.004	< 0.001
Male sex	0.228 (0.225, 0.230)	0.001	< 0.001
Age group	-0.118 (-0.119, -0.117)	0.001	< 0.001
Visit date	-0.0007 (-0.0007, -0.0007)	0.000	< 0.001
Intercept	19.33 (19.24, 19.42)	0.004	< 0.001
**Survival**	**Hazard ratio (95% CI)**		
Intercept (association)	1.262 (1.226, 1.301)	0.015	< 0.001
Adherence status	1.077 (0.616, 1.883)	0.285	0.795
Male sex	1.489 (1.202,1.844)	0.109	< 0.001
Age group	1.369 (1.253, 1.495)	0.045	< 0.001
Visit date	0.998 (0.999, 1.000)	0.000	0.372

Note: Association value represents the effect of change in log VL on time to death

## Discussion

The joint survival model estimated a positive association between the log viral load and time to death as a unit increase in the log viral load was associated with a 26% increase in the risk of death with a hazard rate estimate of 1.262 (95% CI, 1.226, 1.301). A study by Mchunu et al. [14] had similar findings despite using CD4 as the longitudinal profile instead of viral load, whereby it revealed that an increase in CD4 was associated with a 21% reduction in the risk of death to patients. The results are further supported by evidence from a study by Temesgen et al. [13] which showed that CD4 was associated with survival risk but with a focus on TB co-infection. It should be noted that when viral load is suppressed the CD4 count increases. This indicates that higher log viral loads are associated with an increased risk of death for the patient. The finding underscores the significance of viral load control in managing patient outcomes and suggests that effective viral suppression may lead to improved survival rates.

The joint survival model’s estimates for the association between time to death and age group revealed that a 10-year increase in age was associated with a 37% increase in the risk of death. These results are similar to those of other studies conducted in different countries using the joint survival model [15, 16]. This finding highlights the well-known association between advancing age and mortality risk, emphasizing the need for age-specific care and interventions to address the unique challenges faced by older patients.

Being male was associated with an increase in the risk of dying by 49% compared to females. These results are similar to the study conducted in Ethiopia by Khorashadizadeh et al. [15], which showed that men had a higher hazard of death using a joint latent class model (JLCM) which is an extension of the joint survival model. In addition, these results are supported by the findings from studies conducted in Ethiopia, Sri Lanka, and Iran, which used a similar proportional hazards model but used CD4 instead of viral load as a longitudinal trajectory [18–20]. The results however differ from those by Tiruneh et al. [16] Tiruneh et al. (2021)conducted in Ethiopia that revealed that females had higher risk as compared to men. This finding suggests that sex is an important factor influencing survival outcomes, and it may warrant further investigation to understand the underlying reasons for this disparity.

## Conclusion

The joint survival model analysis provided valuable insights into the associations between time to death and viral with the age and sex of the patients. The positive association between log viral load and mortality highlights the critical role of effective viral load suppression in improving patient outcomes. This underscores the importance of regular monitoring and timely intervention to achieve and maintain viral load suppression. The consistent association between age and mortality reinforces the need for age-specific care and interventions to address the unique needs of older patients. Understanding the factors that influence time to death in patients is crucial in improving patient care. Healthcare providers should consider age as a key factor in treatment decision-making and follow-up care.

### Limitations of the study

The study limitations include the unavailability of an important ART regimen variable which is a potential confounder hence its effect could not be captured. Also, the presence of missing values is another limitation as it resulted in the reduction of the observations during the analysis. Another limitation is that earlier years’ viral load measurements were few; however, this was compensated for in this analysis by including other covariates. Hence, we suggest further studies that account for missing values and biased data as well as accounting for ART regimen. Finally, the use of secondary institutional data has some limitations because some variables that could be important confounders were not available; future studies could use a well-designed cohort but over a longer period because the events of interest can be very few for a smaller sample size of participants.

## Data Availability

The datasets analyzed in this study are not publicly available because they are primarily the property of the Ministry of Health of the United Republic of Tanzania. The data, however, are available on reasonable request from the Ministry of Health (Tanzania) through its agency the National AIDS Control Programme.

## Abbreviations

AIDS: Acquired immunodeficiency syndrome
CI: Confidence Interval
HIV: Human Immunodeficiency Virus
HR: Hazard Ratio
ICC: Intra–class correlation
JLCM: Joint Latent Class Model
LMM: Linear Mixed Model
NACP: National AIDS Control Programme
NIMR: National Institute for Medical Research
RR: Risk Ratio
TB: Tuberculosis
THIS: Tanzania HIV Impact Survey
